# Targeting PD-L1 in non-small cell lung cancer using CAR T cells

**DOI:** 10.1038/s41389-020-00257-z

**Published:** 2020-08-13

**Authors:** Ming Liu, Xu Wang, Wei Li, Xinfang Yu, Pedro Flores-Villanueva, Zijun Y. Xu-Monette, Ling Li, Mingzhi Zhang, Ken H. Young, Xiaodong Ma, Yong Li

**Affiliations:** 1 National Clinical Research Center for Respiratory Disease, State Key Laboratory of Respiratory Disease, Guangzhou Institute of Respiratory Health, The First Affiliated Hospital of Guangzhou Medical University, 510120 Guangzhou, China; 2 Department of Medicine, Baylor College of Medicine, Houston, TX 77030 USA; 3 Department of Pathology, Division of Hematopathology, Duke University Medical Center, Durham, NC USA 27708; 4 Department of Oncology, The First Affiliated Hospital of Zhengzhou University, Lymphoma Diagnosis and Treatment Center of Henan Province, 450052 Zhengzhou, China; 5 Institute for Brain Research and Rehabilitation, South China Normal University, 510631 Guangzhou, China

**Keywords:** Cancer, Cell biology

## Abstract

Antibodies against programmed cell death protein 1 (PD-1) and its ligand (PD-L1) have dramatically changed the landscape of therapies for non-small cell lung carcinoma (NSCLC); however, the majority of patients do not respond to these agents. In addition, hyperprogressive disease (HPD) develops in a larger portion of NSCLC patients treated with PD-1/PD-L1 inhibitors than in patients treated with standard chemotherapy. The use of chimeric antigen receptor (CAR) T cells has been successful to treat blood cancers but not for solid tumors like NSCLC. In this work, we constructed CAR T cells that target PD-L1 and evaluated their efficacy in NSCLC with either high or low PD-L1 expression. PD-L1-CAR T cells exhibited antigen-specific activation, cytokine production, and cytotoxic activity against PD-L1^high^ NSCLC cells and xenograft tumors. Furthermore, the addition of a subtherapeutic dose of local radiotherapy improved the efficacy of PD-L1-CAR T cells against PD-L1^low^ NSCLC cells and tumors. Our findings indicate that PD-L1-CAR T cells represent a novel therapeutic strategy for patients with PD-L1-positive NSCLC, particularly for those who are susceptible to HPD.

## Introduction

Lung cancer is the leading cause of cancer-related death in the world^1^. In the United States, approximately 234,000 lung cancer cases are diagnosed with 154,050 deaths annually^1,2^. Approximately 85% of patients with lung cancers have non-small cell lung carcinoma (NSCLC), and more than 40% of them are diagnosed with metastatic disease^1,3^. Although significant progress in drug development against lung cancer has been made, the prognosis of lung cancer has not improved drastically over the past three decades^4^. Even with the current targeted therapies, most patients eventually experience disease relapse^5,6^.

Immunotherapy is a promising therapeutic approach for patients with refractory cancers. Checkpoint inhibitors that target programmed cell death protein 1 (PD-1) or its ligand (PD-L1) have demonstrated efficacy and safety in patients with NSCLC and are becoming a standard treatment for the management of locally advanced and metastatic lung cancer. However, only approximately 20% of unselected patients with advanced NSCLC benefit from this treatment^7^. Furthermore, NSCLCs harboring epidermal growth factor receptor (*EGFR*) mutations or anaplastic lymphoma receptor tyrosine kinase (*ALK*) rearrangements are associated with low overall response rates to PD-1/PD-L1 blockade^8,9^. In addition, hyperprogressive disease (HPD) represents a new pattern of progression that was recently described in cancer patients treated with PD-1/PD-L1 inhibition. A recent report shows that anti-PD-1/PD-L1 treatment accelerates tumor progression in 16% of patients with NSCLC across multiple histologies^10,11^. A potential mechanism is that the fragment crystallizable (Fc) receptor of tumor-associated macrophages engages with the Fc region of the anti-PD-1 antibody to induce HPD^12^. Furthermore, patients with cancers that harbor mouse double minute 2 homolog (*MDM2*) amplification or *EGFR* mutations have increased the risk of HPD after anti-PD-1/PD-L1 treatment^13^. Therefore, there is an urgent need for alternative approaches to target PD-L1-positive tumors in NSCLC patients at high risk of HPD.

Chimeric antigen receptor (CAR) T-cell therapy has been successfully employed in blood tumors but not in solid tumors. The tumor microenvironment generated by myeloid-derived suppressor cells; regulatory T cells; immunosuppressive cytokines, such as interleukin (IL)-10 and transforming growth factor-β; and ligands for tumor-expressed T-cell inhibitory signaling receptors, such as PD-1 and CTLA-4, contribute to attenuated persistence and antitumor efficacy of CAR T cells in solid tumors^14,15^. The addition of checkpoint inhibitors has been applied to enhance CAR T cell efficacy^16^. It is shown that PD-L1 on tumor cells or on dendritic cells and macrophages in the tumor microenvironments exerts functionally significant suppressive effects on tumor immunity^17–19^.

High expression of PD-L1 has been found in cancer cells of NSCLC patients, and CAR T cells that secrete the anti-PD-L1 antibody have demonstrated promising efficacy in humanized mouse models^20–22^. In this study, we demonstrated that PD-L1-CAR T cells have substantial antitumor activity in vitro and lead to prolonged remission for PD-L1^high^ NSCLC xenograft tumors in mice. In addition, radiotherapy exhibited synergistic activity with PD-L1-CAR T cells, potentially by allowing the migration of CAR T cells to tumors generated from PD-L1^low^ NSCLC cells. Our findings provide preclinical evidence to support PD-L1 targeting by CAR T cells to treat NSCLC and potentially other types of solid malignancies.

## Material and methods

### Cell lines and culture

Human NSCLC *EGFR-*wild type cell lines A549 and H1299, *EGFR*-mutant cell lines HCC827 (del E746-A750) and H1975 (L858R and T790M), and normal bronchial epithelial cell line (BEAS-2B) were purchased from ATCC (Manassas, VA). The NSCLC *EGFR*-mutant cell line PC9 (del E746–A750) was obtained as described previously^23^. These cell lines were maintained in RPMI-1640 (Gibco, Gaithersburg, MD) supplemented with 10% heat-inactivated fetal bovine serum (Gibco) and 1% (v/v) penicillin/streptomycin in a humidified incubator with 5% CO_2_ at 37 °C. All cells were transduced with firefly luciferase (Fluc) via lentiviral transduction, and blasticidin selection was utilized to set up stable luciferase-expressing cell lines.

### CAR construction, lentiviral vector production, and T cell transduction

The PD-L1-CAR, encoding single-chain variable fragment (scFv) against the human PD-L1, a CD8 hinge and transmembrane domain, 4-1BB co-stimulatory domain, and CD3ζ signaling domain, were totally synthesized and cloned into a third-generation lentiviral plasmid backbone with a human elongation factor 1α (EF-1α) promoter. PD-L1 scFv is derived from atezolizumab, a fully humanized, engineered monoclonal antibody of IgG1 isotype against PD-L1 (sold as Tecentriq® by Roche). A CD19-CAR with the same structure was used as a control. CD19 scFv is derived from mouse monoclonal antibody FMC-63 (GenBank ID: HM852952.1). PD-L1-CAR-encoding and CD19-CAR-encoding lentiviral supernatants were produced via transient transduction of the 293T cell line as described^24^. CD3^+^/CD4^+^/CD8^+^ T cells were isolated from leukopaks of healthy volunteer donors (Gulf Coast Regional Blood Center, Houston, TX) using EasySep™ Human CD3/CD4/CD8 Positive Selection Kit (Stem Cell Technologies, Vancouver, Canada). Isolated human T cells were then activated by anti-CD3/CD28 beads (Life Technologies, Carlsbad, CA) in a cell-to-bead ratio of 1:3 with 200 IU/ml IL-2 in CTS™ OpTmizer™ T Cell Expansion medium (Life Technologies). After 24 h, activated T cells were transduced by the PD-L1-CAR or CD19-CAR. Medium with IL-2 was refreshed every 2–3 days. Each ensuing cellular or animal experiment was performed using T cells from at least two different donors. In all figure legends, the data were obtained using T cells from one donor only with technical triplicates.

### Cellular cytotoxicity assay

The cytotoxicity of T cells was assessed using a luciferase-based assay as previously described^25^. Stable Fluc-expressing tumor cells (20,000 cells per well) were co-incubated with PD-L1- or CD19-CAR T cells for 4 or 20 h at effector-to-target (E:T) ratios from 10:1 to 1:4. The one-step glow assay kit (Thermo Fisher Scientific, Waltham, MA) was used to measure residual luciferase activity from the remaining tumor target cells, and lysis was calculated as follows: % lysis = 100 – (Fluc from CAR-T-treated wells) / (Fluc from untreated target cells) × 100.

### Cytokine secretion assay

Cytokine production by CAR T cells in vitro was evaluated following the co-incubation of CAR T cells with tumor cells at a 2:1 ratio for 20 h. Supernatants were harvested, and cytokine levels were measured using Human DuoSet ELISA kits (IL-2, tumor necrosis factor [TNF]-α, and interferon [IFN]-γ, R&D Systems, Minneapolis, MN).

### Flow cytometry

Expression levels of PD-L1 and other cell surface markers on tumor cells and T cells were measured using flow cytometry. CAR T cells were collected from cultures and detected with monoclonal antibodies against human CD3, CD4, CD8, TIM3, CD45RA, CD62L, PD-L1, and PD-1 (Biolegend, San Diego, CA) according to the manufacturers’ instructions. PD-L1-CAR expression was detected using an indirect method with biotinylated protein L and a streptavidin-coupled PE antibody (Becton Dickinson, Franklin Lakes, NJ)^26^. Fluorescence was assessed using BD Accuri™ C6 Plus or LSRFortessa instruments (BD Biosciences, San Jose, CA), and the data were analyzed using FlowJo v10 (Tree Star, Ashland, OR).

### Xenograft mouse model

All animal procedures were performed in accordance with our Institutional Animal Care and Use Committee requirements under an approved protocol. Female NOD.Cg-Prkdc^scid^ Il2rg^tm1Wjl^/SzJ (NSG, The Jackson Laboratory, Bar Harbor, ME) mice aged 6–8 weeks were maintained in a pathogen-free barrier facility. Mice were inoculated subcutaneously with 1.0 × 10^6^ H1975-Fluc cells, 3 × 10^6^ HCC827-Fluc cells, 5.0 × 10^6^ A549-Fluc cells, or 2.0 × 10^6^ H1299-Fluc cells. Animals were treated with 5 × 10^6^ CAR T cells twice via tail vein injection on day 7 and 10 post-tumor cell inoculation. Tumor progression was monitored using an IVIS Spectrum in vivo Imaging System (Perkin Elmer, Waltham, MA). Tumor volumes were calculated according to the formula: *V* = ½ (length × width^2^).

### Histology immunohistochemistry analyses

Tissue samples were collected and stained followed the manufacturer’s protocol. In brief, deparaffinized and rehydrated sections were treated for antigen retrieval using sodium citrate buffer, blocked with normal goat serum for 30 min at room temperature, and then incubated with primary antibody against CD3 (ab16669, Abcam, Cambridge, UK), PD-L1(ab228462, Abcam), Ki67(D2H10, Cell Signaling Technology, Danvers, MA) at 4 °C overnight. Slides were incubated with secondary antibodies, counterstained with hematoxylin, and visualized by the Ultra Vision Detection System (Thermo Fisher Scientific). Signal intensity was scored by two independent observers who were blind to the experimental groups.

### Statistical analysis

The data were presented as means ± standard error (SEM). Two independent groups were analyzed using Student’s *t*-test, while the statistical comparison between multiple groups was performed using two-way repeated-measures ANOVA. *p*-values ≤ 0.05 were considered statistically significant. All statistical analyses were performed using GraphPad Prism v7.0 (GraphPad Software, San Diego, CA).

## Results

### Generation of PD-L1-CAR-expressing T cells

**Fig. 1:**
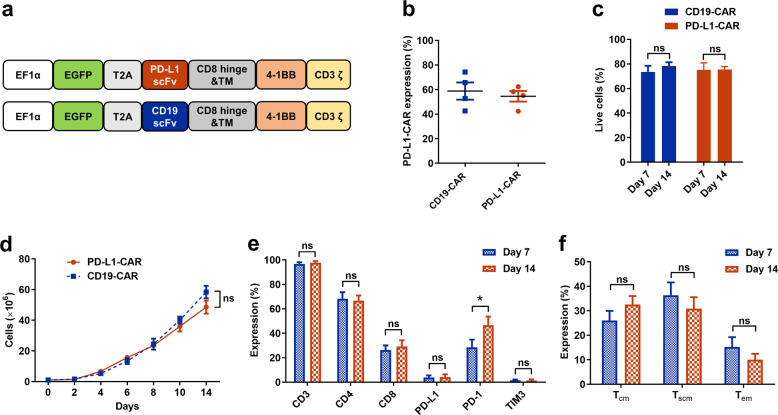
Characteristics of PD-L1-CAR T cells. **a** Schematic diagram of PD-L1-CAR and CD19-CAR constructs. scFv for PD-L1 is derived from atezolizumab (Roche). **b** Surface expression of PD-L1-CAR on transduced T cells as measured by flow cytometry using Biotin-Protein L and APC-streptavidin on day 5 post transduction. **c** Viability of PD-L1-CAR T cells on day 7 and 14 post-transduction. **d** Expansion of PD-L1-CAR and CD19-CAR T cells in vitro for 14 days. **e** Percentage of CAR T cells that were positive for CD3, CD4, CD8, PD-1, PD-L1, and TIM3 on day 7 and 14. **f** Percentage of CAR T cells that were positive for memory cell markers on day 7 and 14. Data represented technical triplicates using T cells from one donor and were displayed as mean ± SEM. *p ≤ 0.05, ns not significant.

To generate anti-PD-L1-CAR T cells, we constructed a CAR expression plasmid that encodes an anti-PD-L1 scFv, a CD8 hinge, a transmembrane domain in tandem with 4-1BB intracellular signaling domain, and a CD3ζ motif (Fig. 1a). A CD19-CAR with an anti-CD19 scFv was used as a control. The expression level of the CAR was evaluated by flow cytometry 5 days of post-transduction. An average of 55.6% of the PD-L1-CAR T cells and 59.3% of the CD19-CAR T cells were scFv-positive, indicating a high transduction efficiency (Fig. 1b, Supplementary Fig. 1a). There was no significant difference in cell viability between PD-L1-CAR T and CD19-CAR T cells on day 7 and 14 post-transduction (Fig. 1c). CD19-CAR and PD-L1-CAR T cells displayed a similar expansion tendency after priming with anti-CD3/CD28 beads for 14 d (>50-fold expansion; Fig. 1d). Importantly, both CD4^+^ and CD8^+^ PD-L1-CAR T cells, upon anti-CD3/CD28 bead stimulation, were expanded to more than 5 × 10^7^ cells from an initial 1 × 10^6^ transduced primary T cells (Supplementary Fig. 2a). To investigate the expression level of different cell markers at an early and late stage of culture, we examined T-cell markers for lineage, immunosuppression, and memory phenotypes. On day 7, both PD-L1-CAR and CD19-CAR T cells were positive for CD3 (96.8 and 97.8%, respectively), CD4 (68.3 and 66.8%, respectively), and CD8 (26.4 and 29.1%, respectively). For PD-L1-CAR T cells, 28.5%, 3.9%, and 1.4% of them were positive for PD-1, PD-L1, and TIM3, respectively, on day 7. During PD-L1 CAR T cell expansion, the expression of PD-1 on these cells was higher at day 14 than that at day 7 (Fig. 1e, Supplementary Fig. 1b); however, there was no differences in fractions of central memory T cells (CD45RA^−^CD62L^+^, *T*_cm_), stem cell-like memory T cells (CD45RA^+^CD62L^+^, *T*_scm_), and effector memory T cells (CD45RA^+^CD62L^−^, *T*_em_) between day 14 and day 7 (Fig. 1f, Supplementary Fig. 1c). In summary, after 14 days of culture, CD3^+^, CD4^+^, and CD8^+^ PD-L1-CAR T cells expanded well and contained both effector and central memory cell populations.

### PD-L1-CAR T cells exhibit robust effector functions against PD-L1^high^ NSCLC cells in vitro

**Fig. 2:**
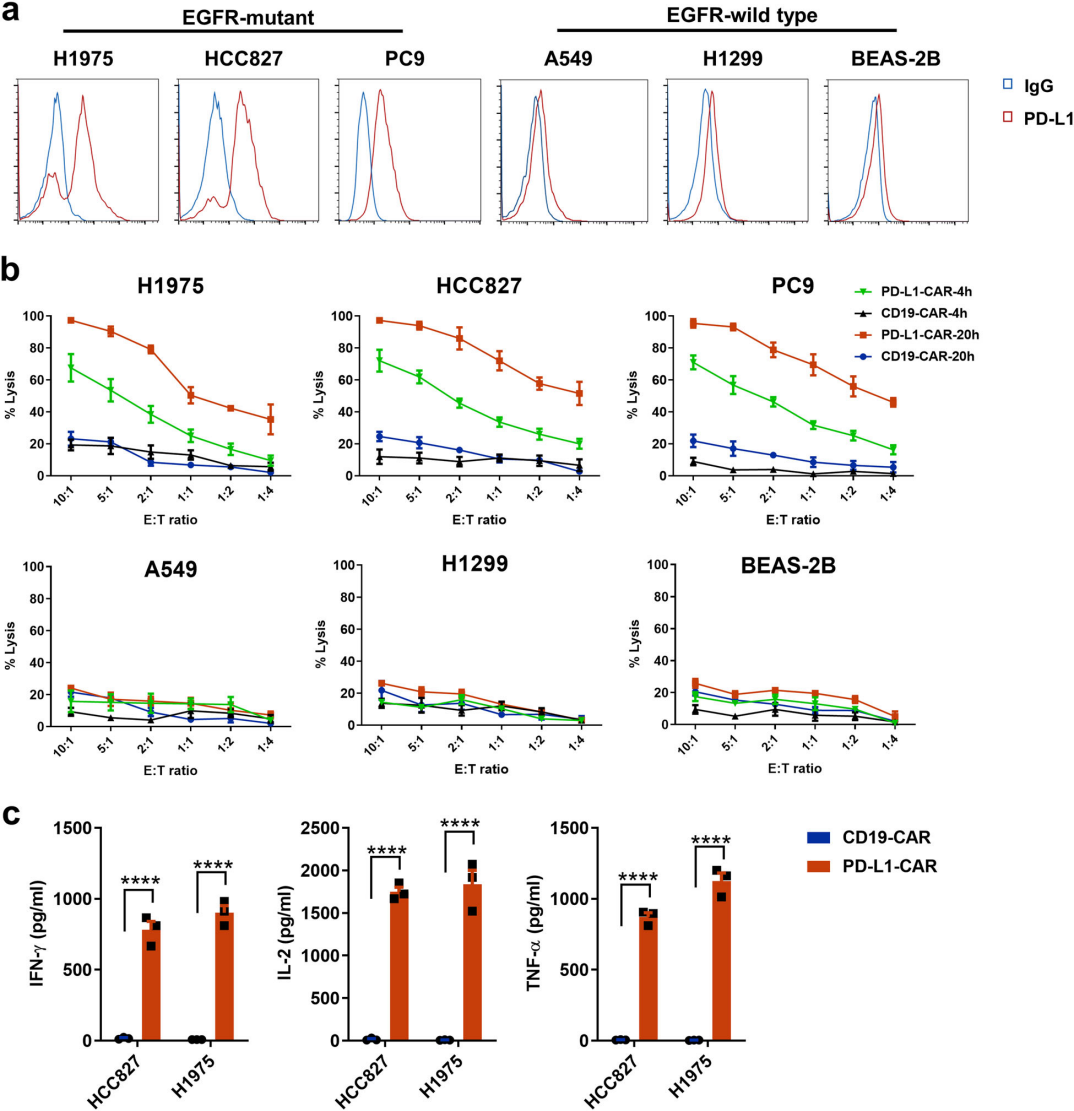
PD-L1-CAR T cells kill NSCLC cells in a PD-L1-dependent manner. **a** Flow cytometry histogram of the surface antigen expression of PD-L1 in human NSCLC cell lines and BEAS-2B. **b** Cytotoxic activity of PD-L1-CAR T cells after 4 and 20 h of co-culture with human NSCLC cell lines and BEAS-2B. PD-L1-CAR T cells and CD19-CAR T cells were used as effector cells at various ratios of effector (E): target (T). **c** Secretion of cytokines analyzed by ELISA in supernatants obtained after a 20-h co-culture of effector and target cells at a 2:1 E:T ratio. Data represented technical triplicates using T cells from one donor and were shown as mean ± SEM. ****p ≤ 0.0001.

PD-L1 was highly expressed on H1975, HCC827, and PC9 cells, which carry mutant *EGFR*, whereas A549, H1299 (both with wild-type *EGFR*), and an immortalized bronchial cell line (BEAS-2B) showed lower PD-L1 expression (Fig. 2a). To assess the antitumor efficacy of PD-L1-CAR T cells, we performed cytotoxicity assays against these NSCLC cell lines and BEAS-2B. PD-L1-CAR T cells and CD19-CAR T cells were co-cultured at selected effector-to-target ratios for 4 or 20 h. Compared to CD19-CAR T cells, PD-L1-CAR T cells showed significantly stronger cytotoxic activity for PD-L1^high^ cell lines (H1975, HCC827, and PC9) but not for PD-L1^low^ cell lines (A549, H1299, and BEAS-2B; Fig. 2b). Furthermore, both CD4^+^ and CD8^+^ PD-L1-CAR T cells exhibited efficient cytotoxicity against PD-L1^high^ tumor cells (Supplementary Fig. 2b). PD-L1-CAR T cells also demonstrated antigen-specific production of cytokines IL-2, IFN-γ, and TNF-α when incubated with PD-L1^high^ tumor cell lines HCC827 and H1975 in vitro (Fig. 2c).

### PD-L1-CAR T cells eradicate PD-L1^high^ NSCLC tumors in vivo

**Fig. 3:**
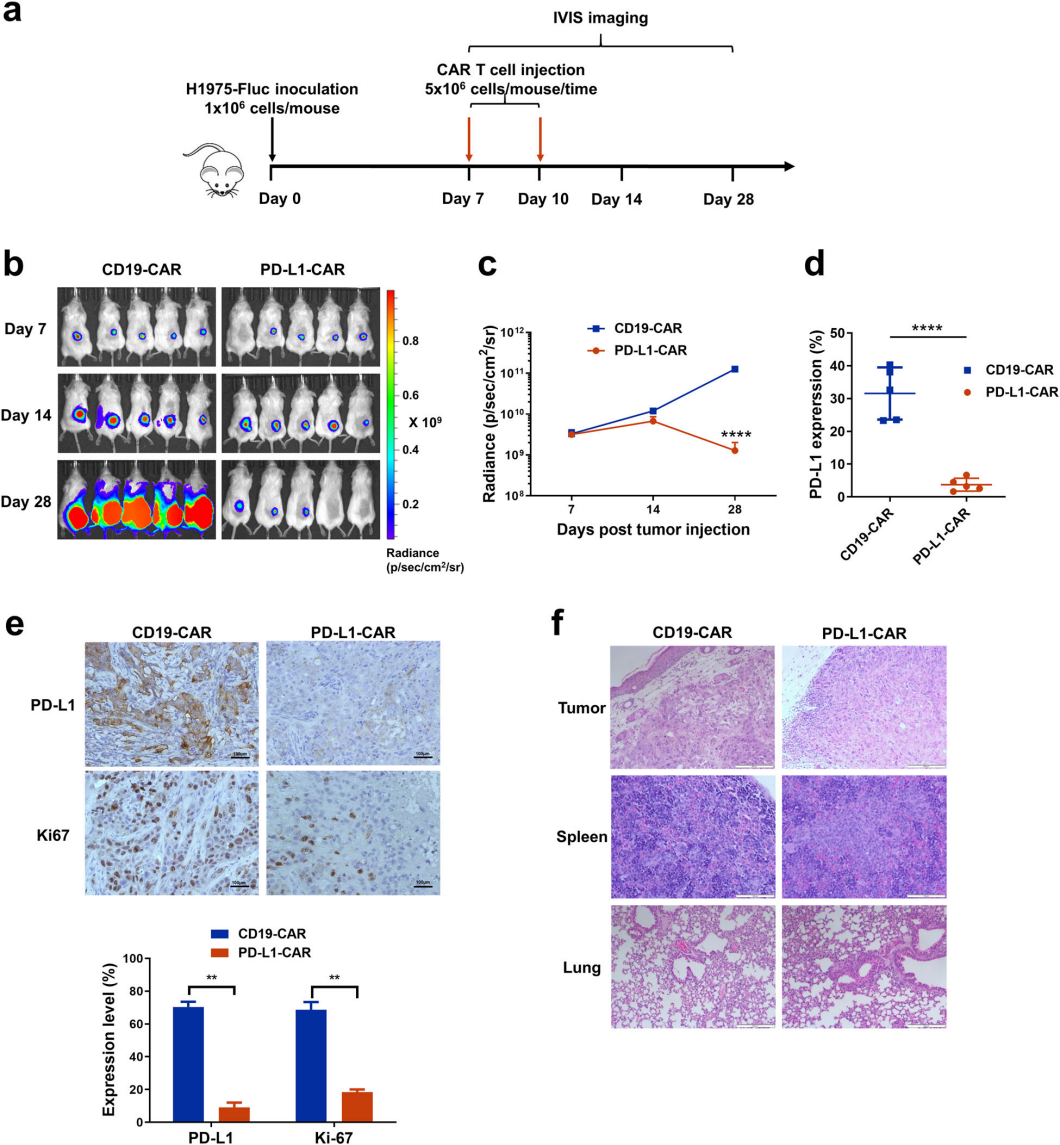
PD-L1-CAR T cells inhibit the growth of human PD-L1high NSCLC in a xenograft model. **a** Experimental design of the tumor xenograft model infused with PD-L1-CAR or CD19-CAR T cells. **b**.NSG mice were inoculated with 1.0 × 10^6^ H1975-Fluc cells and infused intravenously with 5 × 10^6^ PD-L1-CAR T cells or CD19-CAR T cells twice on day 7 and 0 (*n* = 5 mice per group). Bioluminescence imaging was used to assess tumor growth on day 7, 14, and 28 post tumor cell inoculation. **c** Bioluminescence kinetics of H1975-Fluc (*n* = 5 mice per group). **d** The percentage of PD-L1-positive cells within tumors. All cells were extracted from tumors of each treatment group on day 28 after inoculation and the expression of PD-L1 was evaluated by flow cytometry. **e** Representative IHC images of CD19-CAR or PD-L1-CAR T cell-treated NSCLC tumors for PD-L1 and Ki67. Scale bars, 100 µm. **f** Hematoxylin and eosin staining of tumors or organs on day 28. Scale bars, 200 µm. ****p ≤ 0.0001.

To determine the efficacy of PD-L1 CAR T cells in vivo, we inoculated NSG mice subcutaneously with H1975-Fluc followed by two doses of PD-L1-CAR or CD19-CAR T cells via tail vein on day 7 and 10. Tumor xenografts were monitored via bioluminescence imaging weekly (Fig. 3a). Serial imaging of luminescence showed that PD-L1-CAR T cells dramatically decreased tumor burden compared with CD19-CAR T cells (Fig. 3b). The radiance of the tumors was significant reduced after PD-L1-CAR T-cell treatment (Fig. 3c). Flow cytometry analysis on cells extracted from tumors day 28 post-inoculation indicated that the expression of PD-L1 was significantly decreased in tumor cells upon PD-L1-CAR T cell treatment (Fig. 3d). This result was corroborated by immunohistochemical (IHC) analysis of PD-L1 in tumor sections (Fig. 3e). Fewer Ki67-positive cells were observed in tumors treated with PD-L1-CAR T cells than that with the control CD19-CAR T treatment (Fig. 3e). No significant differences in tissue morphology were found in the spleens and lungs from mice treated with PD-L1-CAR or CD19-CAR T cells (Fig. 3f).

**Fig. 4:**
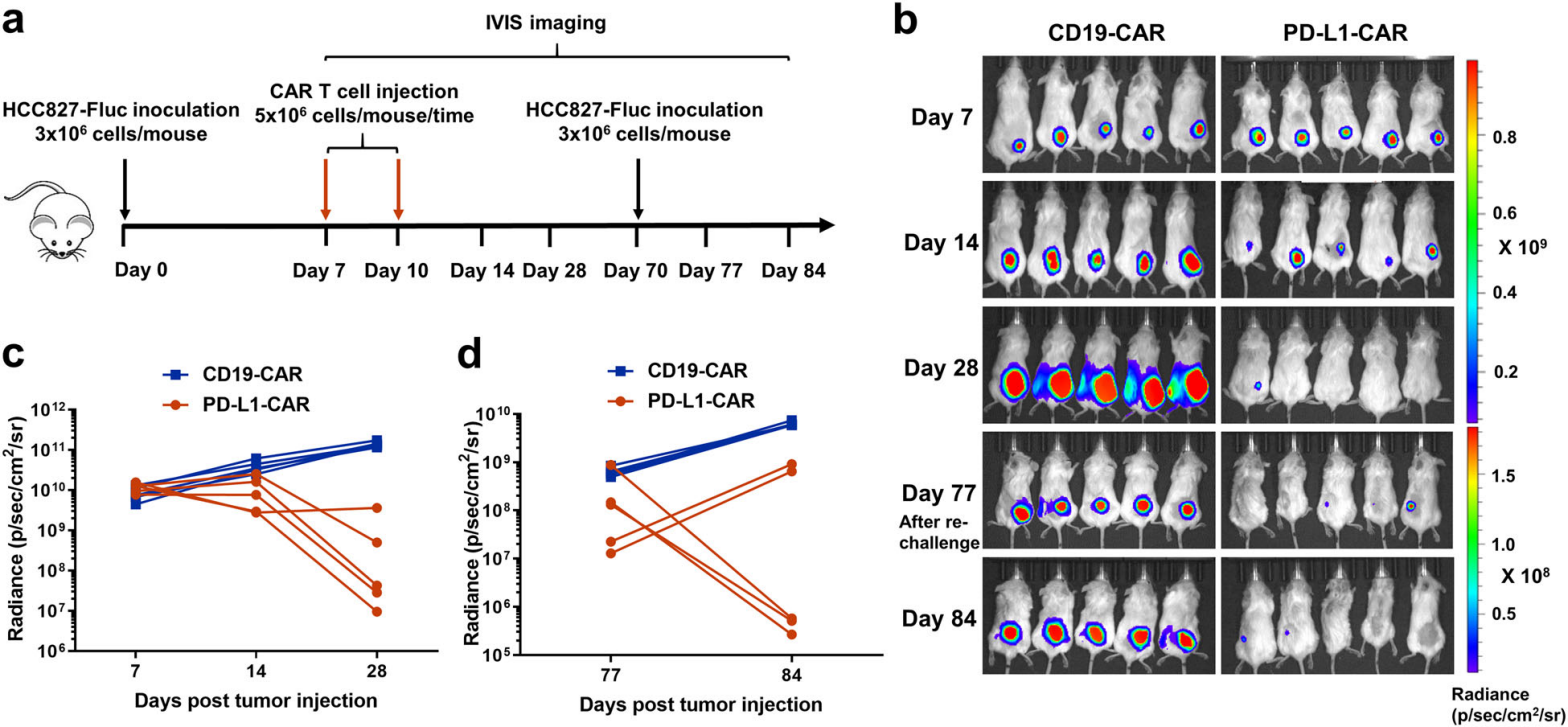
Persistence of PD-L1-CAR T cells in mice. **a** Experimental design of HCC827-Fluc tumors with PD-L1-CAR T-cell therapy and re-challenge. **b** Serial bioluminescence imaging of tumor progression and regression. **c**, **d** Bioluminescence kinetics of HCC827-Fluc tumors (*n* = 5 mice per group). For the CD19-CAR T cell control group, a separated cohort of NSG mice was challenged with HCC-827-Fluc cells at day 70.

We next tested PD-L1-CAR T cells in treating another PD-L1^high^, *EGFR*-mutant tumor cell line HCC827. Tumor xenografts (at the right flank of mice) were monitored via bioluminescence imaging weekly after injection of CAR T cells (Fig. 4a). On day 28, HCC827-Fluc tumor cells were almost completely eliminated by PD-L1-CAR T cell treatment, and no obvious tumor was observed during the next 6 weeks (Fig. 4b, c). The control CD19-CAR T cells exhibited no tumorcidic activity. We re-challenged the mice treated with PD-L1-CAR T cells by injecting another dose of HCC827-Fluc cells on day 70 on the contralateral flank of the animals. Comparable and sustained antitumor activity was observed in the re-challenged group (Fig. 4b, d), while tumor growth was observed in a new control animal cohort treated with CD19-CAR T cells. This suggests that PD-L1-CAR T cells had sustained antitumor activity. Collectively, these results demonstrated that PD-L1-CAR T cells exhibited a significant antitumor effect against PD-L1^high^ NSCLC cells in vivo.

### Evaluation of the role of IFN-γ and irradiation in promoting the antitumor activity of PD-L1-CAR T cells against PD-L1^low^ NSCLC cells

In order to improve the efficacy of PD-L1-CAR T-cell treatment in PD-L1^low^ NSCLC cells, we sought to induce PD-L1 expression in NSCLC cells. We treated cells with 5 ng/ml IFN-γ and analyzed PD-L1 expression. A significant increase in the expression of PD-L1 was observed in all NSCLC cell lines except BEAS-2B (Supplementary Fig. 3a). Next, A549 and HCC827 cells were pre-treated with 5 ng/ml IFN-γ for 24 h followed by PD-L1-CAR T cells or CD19-CAR T cells for an additional 4 or 20 h. We found no significant difference in tumor cell lysis with or without IFN-γ pre-treatment (Supplementary Fig. 3b).

**Fig. 5:**
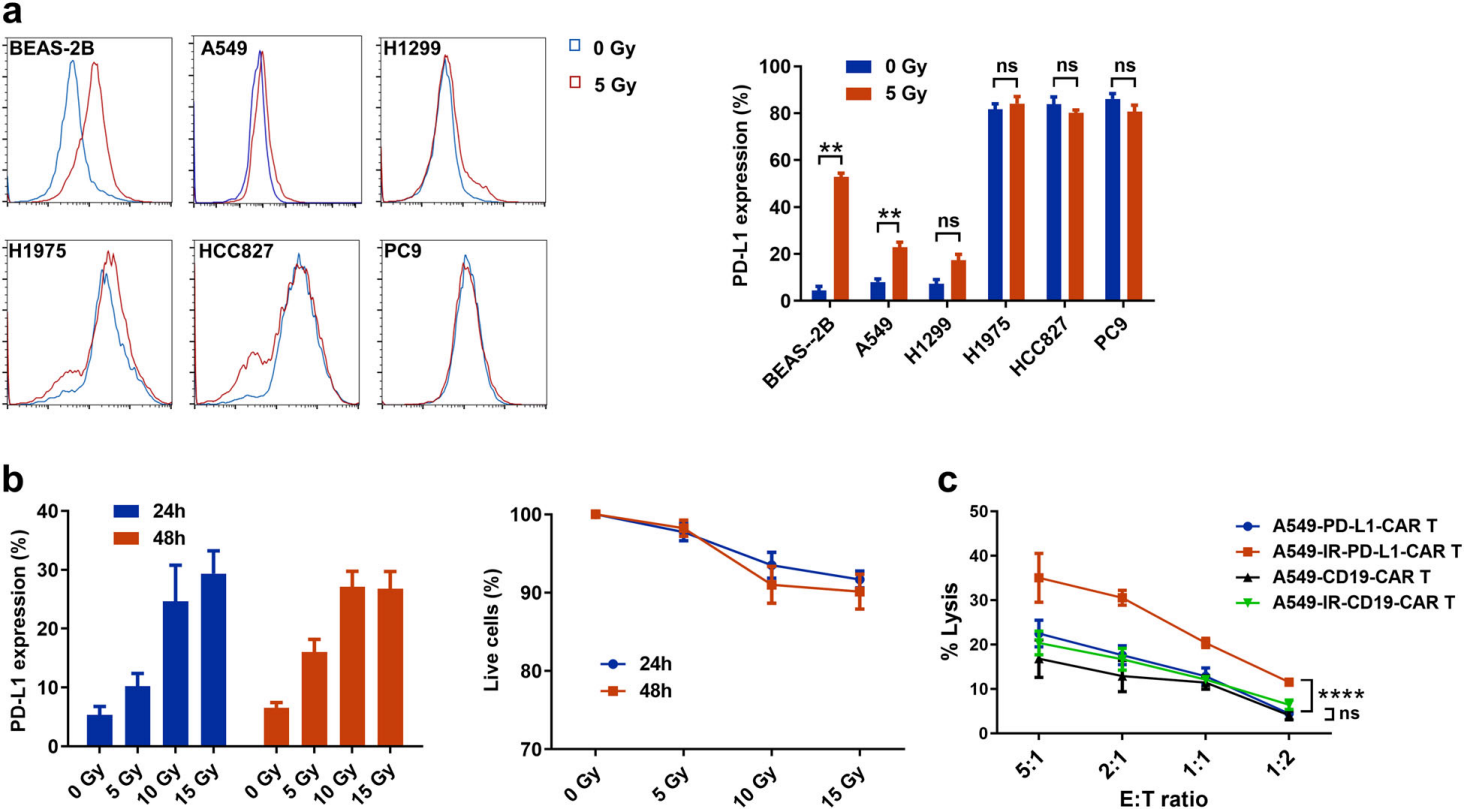
Enhanced tumor PD-L1 expression after irradiation treatment. **a** Signal intensities of PD-L1 expression in cell lines treated with 5 Gy radiation as analyzed by flow cytometry. **b** Percentage of PD-L1-positive cells and cell viability in A549 cells treated with different doses of radiation for 24 or 48 h. **c** The effect of radiation treatment on anti-tumor efficacy of PD-L1-CAR T cells at different effector (E): target (T) ratios. Data represented technical triplicates using T cells from one donor and were shown as mean ± SEM. *p ≤ 0.05, **p ≤ 0.01, ****p ≤ 0.0001, ns not significant.

**Fig. 6:**
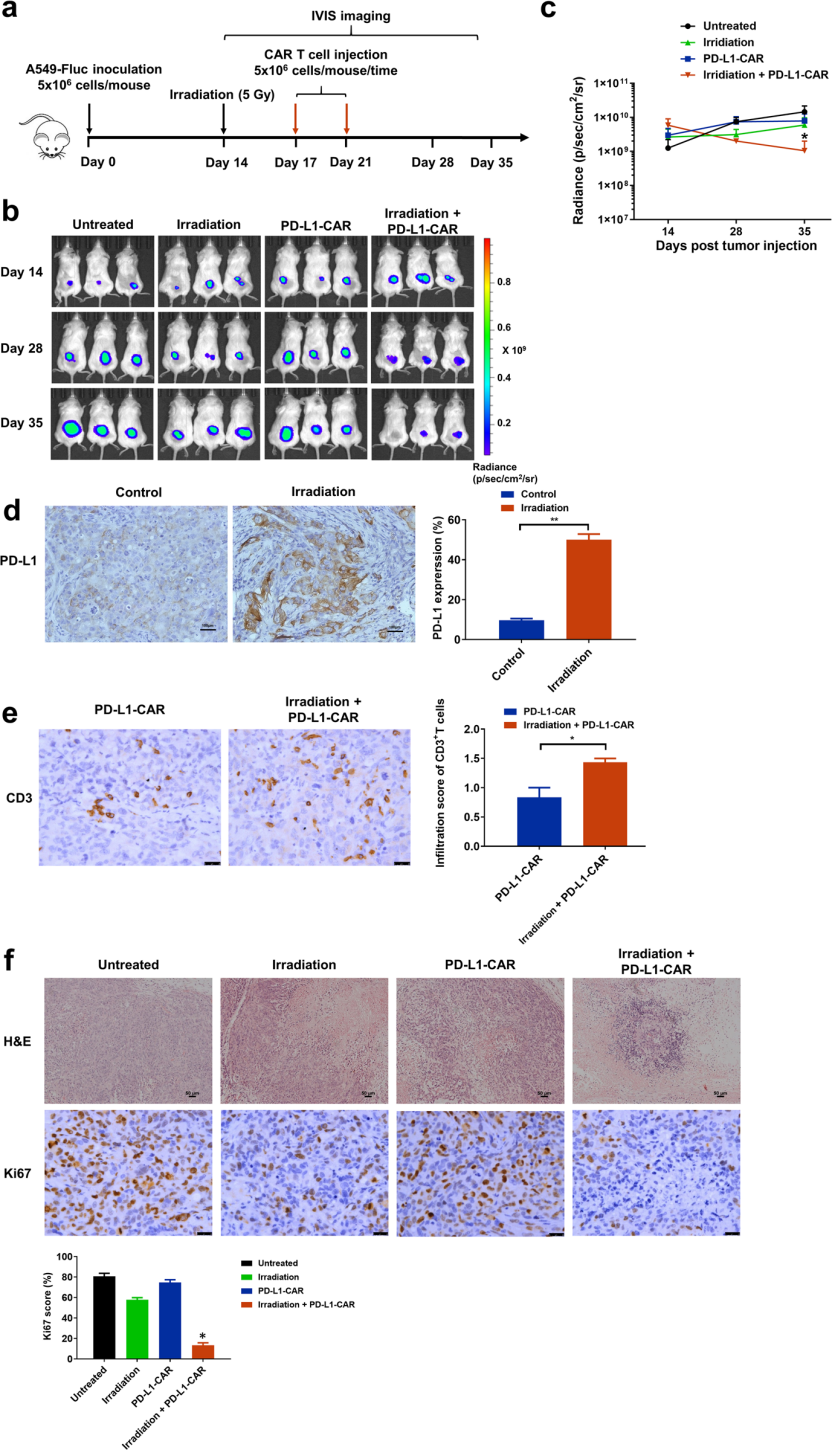
Synergistic efficacy of irradiation and PD-L1-CAR T-cell therapy in a PD-L1 low NSCLC xenograft model. **a** Experimental design of tumor cell xenograft model treated with CAR T cells and/or irradiation. **b** Serial bioluminescence imaging of tumor progression and regression in each group (*n* = 3 mice per group). **c** Bioluminescence kinetics of A549-Fluc (*n* = 3 mice per group) in each treatment group. **d** Representative IHC of PD-L1 in irradiation-treated NSCLC tumors. Scale bars, 100 µm. **e** Representative images of CD3 IHC in PD-L1-CAR T cell-treated and irradiation-treated NSCLC tumors. Scale bars, 100 µm. **f** Hematoxylin and eosin staining of tumors. Scale bars, 50 µm. *p ≤ 0.05.

Radiotherapy, a lung cancer treatment option that directly induces tumor cell apoptotic death and enhances tumor-specific immunity, has been shown to upregulate PD-L1 expression in tumor cells and improve the efficacy of anti-PD-1/PD-L1 therapy^27,28^. Incomplete tumor eradication by radiation-induced adaptive immunity is partially due to the engagement of negative regulatory pathways, such as the PD-L1/PD-1 axis^29^. We applied 5 Gy irradiation to BEAS-2B, A549, H1299, H1975, HCC827, and PC9 cell lines and found moderately and statistically significant increased expression of PD-L1 in PD-L1^low^ A549 cells, but not in H1299 cells (Fig. 5a, b). However, radiation resulted in significant upregulation of PD-L1 in BEAS-2B cells. Irradiation with 5 Gy prior to the addition of PD-L1-CAR T cells significantly increased cytolysis of A549 cells, whereas no significant difference in cytolysis was found with CD19-CAR T cells (Fig. 5c). In mice xenografted with PD-L1^low^ A549 and H1299 cells and then treated with CAR T cells and 5 Gy localized irradiation (Fig. 6a; Fig. S4a), irradiation alone had no effect on tumorigenesis but increased the antitumor activity of PD-L1-CAR T cells (Figs. 6b, c; S4b). Notably, increased PD-L1 expression was observed by IHC in A549 tumors 72 h post-irradiation (Fig. 6d). Irradiation also increased tumor-infiltrating CAR T cells and reduced cell proliferation for both A549 and H1299 tumors (Figs. 6e; S4c). The combination of radiation and PD-L1-CAR-T cells resulted in fewer proliferative tumor cells than either agent alone (Figs. 6f; S4c). The reduction of tumorigenesis for both A549 and H1299 treated by the combination is relatively moderate compared to that for H1975 and HCC827 by CAR T cells alone (Figs. 3, 4). Nonetheless, these data support the notion that the combination of localized irradiation and PD-L1-CAR T cells attenuates the growth of tumors from PD-L1^low^ NSCLC cells.

## Discussion

*EGFR* mutation is a frequent cancer-driving event in NSCLC, occurring in about 40–50% of cases in Asia and 20–30% in the United States^30^. In patients with advanced, *EGFR*-mutant NSCLC, PD-L1 expression is found in more than 50% of cases^31^, but *EGFR*-mutant NSCLC has a poor response to anti-PD-1/PD-L1 treatment^32^. In addition, a significant portion of NSCLC patients develop HPD after anti-PD-1/PD-L1 therapy^10,11^, and the *EGFR* mutation is a proposed risk factor for HPD^13^. In this work, we explored PD-L1-CAR T-cell therapy as an alternative treatment approach for NSCLC with PD-L1^high^ and *EGFR* mutant phenotypes (for example, PD-L1 expression assessed to be ≥50% tumor proportion score). We showed that EGFR-mutant NSCLC cells such as HCC827, H1975, and PC9 expressed high levels of PD-L1 and PD-L1-CAR T cells have strong cytotoxic activity against these cells and xenograft tumors.

PD-L1 is induced in tumors and in cultured tumor cells by IFN-γ exposure. However, in present work, IFN-γ failed to increase PD-L1-CAR T cells efficacy against PD-L1^low^ NSCLC cells. This could be a result of deficiency of IFN-γ treatment dose and duration. Given the transient nature of PD-L1 induction by IFN-γ, future optimization by biologics or compounds should be considered for long-term stimulation of PD-L1 expression without attenuating T cells function. Substantial evidence has shown that the combination of radiotherapy and immunotherapy is more effective than monotherapy^29^. Preclinical studies have demonstrated that PD-L1 expression is upregulated on tumor cells after radiotherapy, resulting in a synergistically enhanced antitumor effect of irradiation and PD-L1 blockade^29^. Patients receiving radiotherapy before anti-PD-1 treatment have a better prognosis than those that receive anti-PD-1 alone. Another study indicated that this synergy stems from type I interferon production induced by radiotherapy^33^. Our results show that radiation improves the killing ability of PD-L1-CAR T cells against NSCLC xenograft tumors that otherwise express low levels of PD-L1. This is likely due to the increased CAR T cell infiltration into the tumors, rather than radiation-mediated elevation of PD-L1 expression on tumor cells. These data could broaden the potential clinical applications of PD-L1-CAR T cells for the treatment of NSCLC and other solid tumors.

Among main difficulties of targeting solid tumors using CAR T cells is the lack of tumor-specific membrane antigens or antigens that are shared by dispensable cell types such as B cells, which prompt many to use suboptimal targets such as PD-L1. Beyond placenta, tonsil, and macrophages in lung and liver, PD-L1 protein is not expressed in steady-state normal human tissues, although the mRNA of *PD-L1* is present in many tissues or cells^34–37^. In mice, CAR T cells targeting Pd-l1 were effective in slowing tumorigenesis in a B16 syngeneic mouse model^38^; the toxicity of CAR T cells towards Pd-l1-expressing mouse tissues was not directly addressed, although Cd11b-positive cells were the most adversely effected lymphocytes by anti-Pd-l1 CAR T cells^38^. In the present study, we only used the NSG model to test the anti-human PD-L1-CAR T cell therapies against human tumors without evaluating the on-target and off-target toxicity in vivo. Radiation may augment the on-target toxicity of PD-L1 CAR T cells in humans as it clearly increases the expression of PD-L1 in BEAS-2B cells. We plan to assess the safety of PD-L1-CAR-T cells by using immunocompetent mouse models before considering phase 1 clinical trials.

In conclusion, PD-L1-CAR T cells are a promising therapeutic strategy for NSCLC with PD-L1^high^ and *EGFR* mutation. Furthermore, the addition of radiation sensitizes PD-L1^low^ *EGFR*-wild type NSCLC to PD-L1-CAR T cells. PD-L1-CAR T cells thus represent a novel therapeutic option for NSCLC patients who are susceptible to HPD.

## Supplementary information

SUPPLEMENTAL MATERIAL
