# Supplementary material for: Targeting PD-L1 in non-small cell lung cancer using CAR T cells
Source: Oncogenesis. 2020 Aug 13;9(8):72. doi: 10.1038/s41389-020-00257-z (PMC7426958; doi:10.1038/s41389-020-00257-z)
Supplement: Supplementary file 1 — SUPPLEMENTAL MATERIAL [file 41389_2020_257_MOESM1_ESM.pdf]

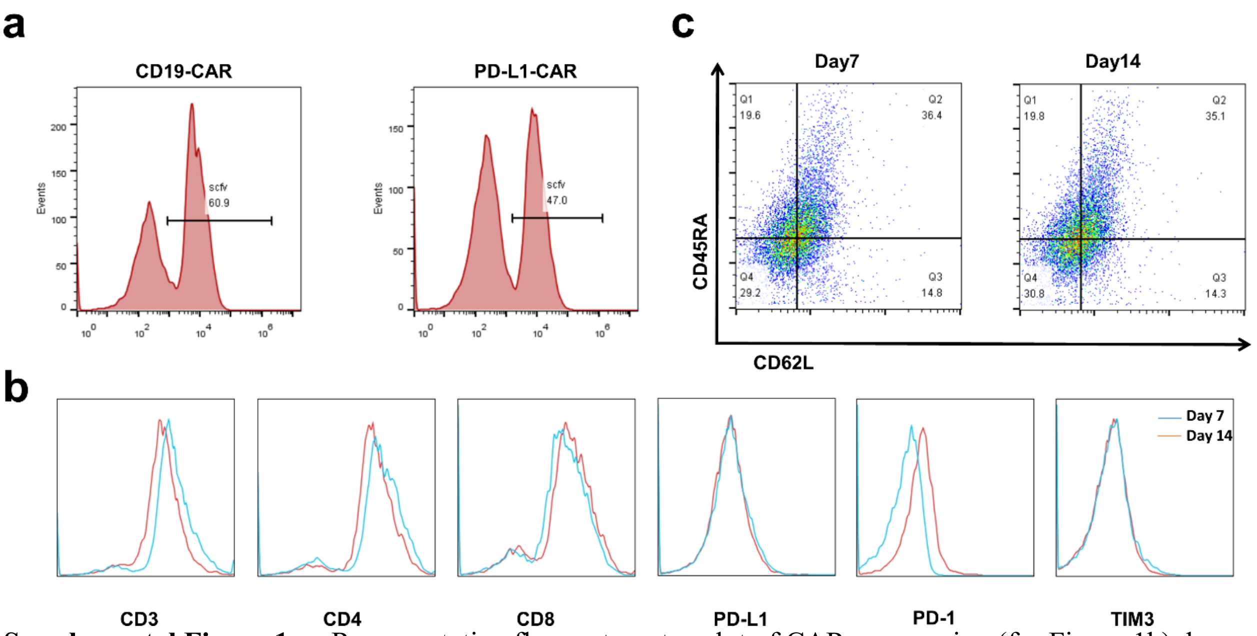

Supplemental Figure 1. a. Representative flow cytometry plot of CARs expression (for Figure 1b). b. Representative flow cytometry of PD-L1-CAR T cells that were positive for CD3, CD4, CD8, PD-1, PD-L1, and TIM3 on day 7 and 14 (for Figure 1e). c. Representative flow cytometry of PD-L1-CAR T cells that were positive for memory cell markers CD62L and CD45RA on day 7 and 14 (for Figure 1f).

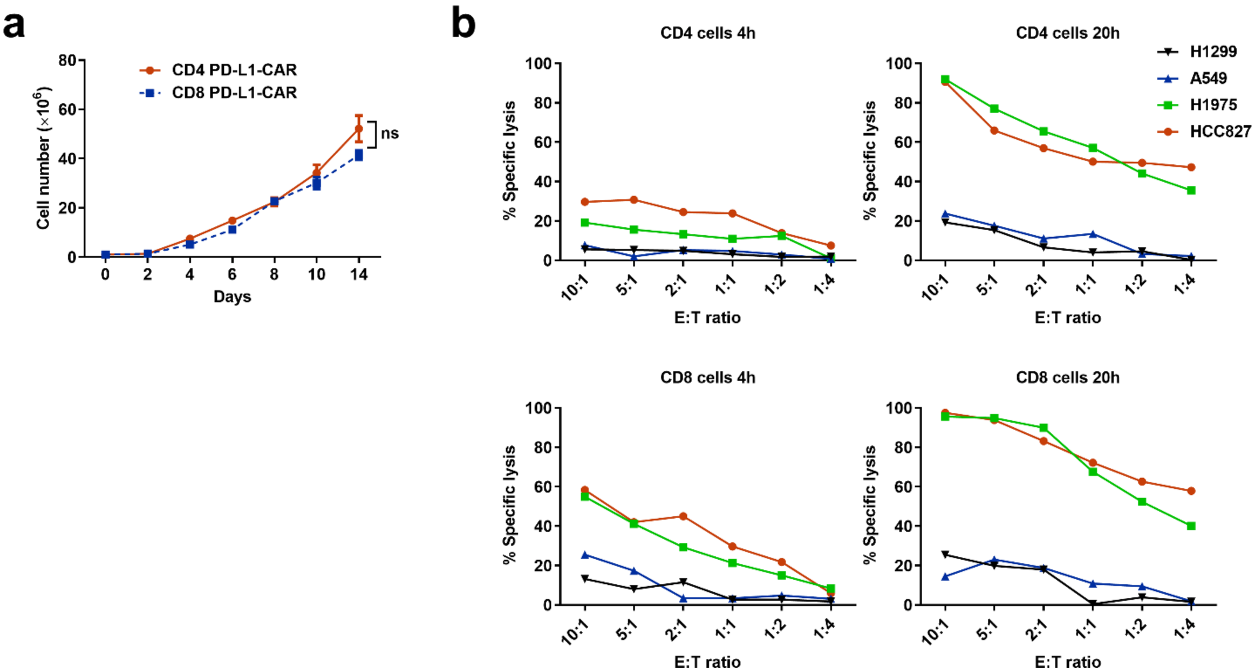

Supplemental Figure 2. CD4/CD8 PD-L1-CAR T cells lyse NSCLC cells in a PD-L1-dependent manner. a. Expansion after 14 d of CD4 and CD8 T cells transduced with CD19-CAR and PD-L1-CAR *in vitro*. b. Cytotoxic activity of CD4 and CD8 PD-L1-CAR T cells after 4 and 20 h of co-culture with NSCLC cell lines. Data represented technical triplicates using T cells from one donor and were displayed as mean ± SEM. ns = not significant.

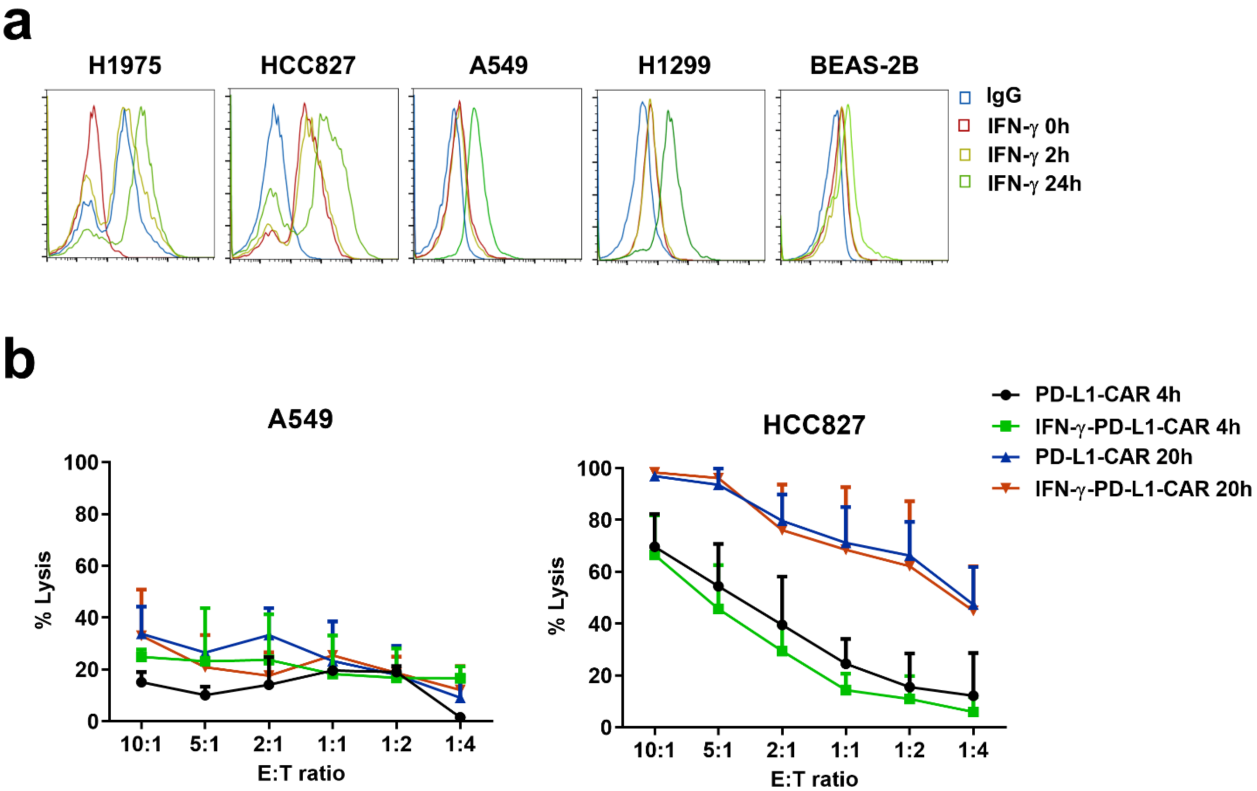

Supplemental Figure 3. Evaluation of IFN-γ with PD-L1-CAR T cells against NSCLC cells. a. PD-L1 expression was measured by flow cytometry in cell lines 2 or 24 h after treatment with 5 ng/mL IFN-γ. b. The anti-tumor efficacy of PD-L1-CAR T cells treated with IFN-γ at different effector (E): target (T) ratios.

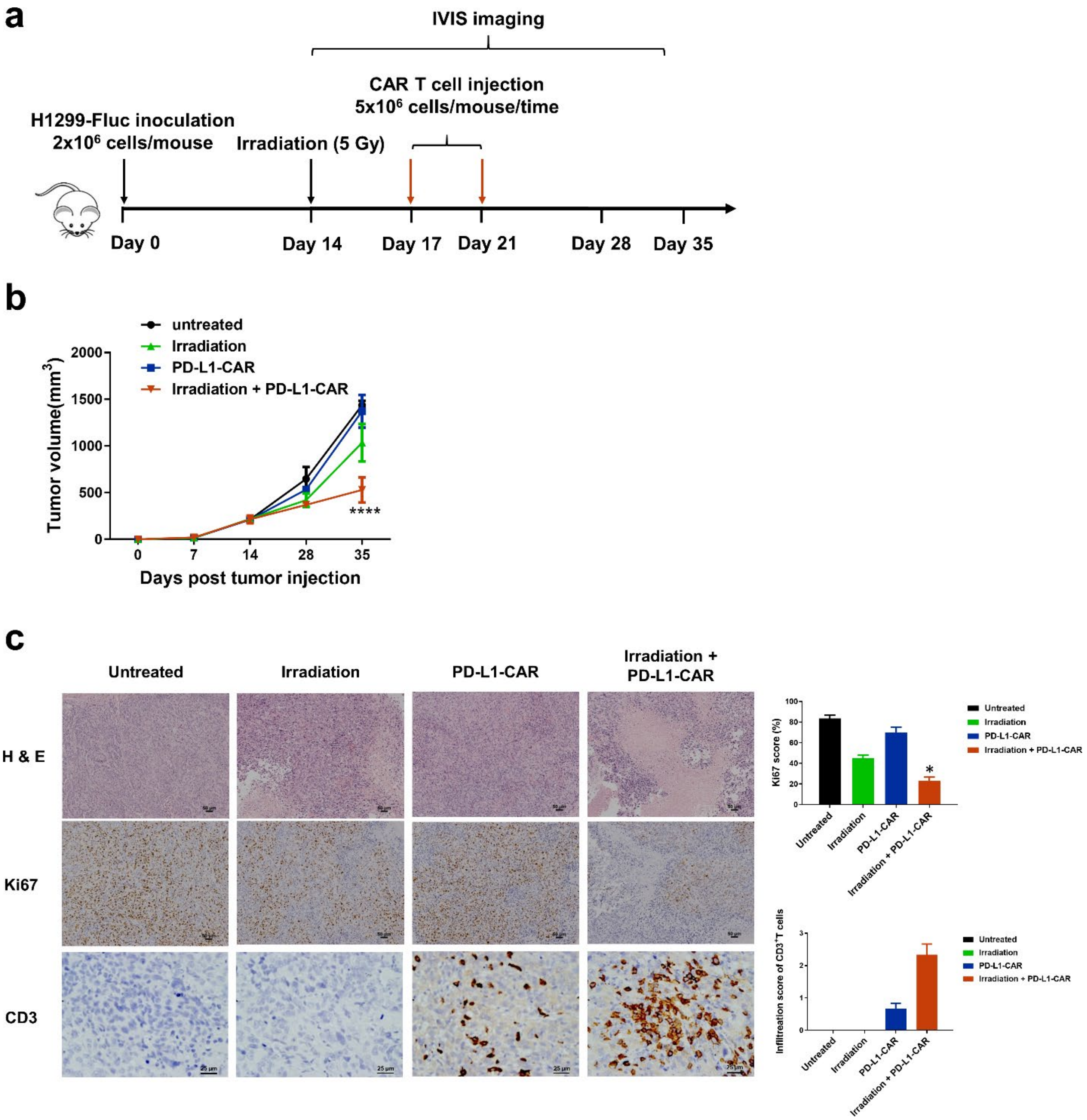

Supplemental Figure 4. Synergistic efficacy of irradiation and PD-L1-CAR T-cell therapy in the H1299 xenograft model. a. Experimental design of tumor cell xenograft model treated with CAR T cells or irradiation. b. Tumor volume in each group of mice (n=3 mice per group). c. H & E staining of the indicated organs. Scale bars, 50 µm. Representative IHC of NSCLC tumors from each group stained for Ki67 and CD3. Scale bars, 50 µm or 25 µm as indicated. **** $p \leq 0.0001$.
